# An Integrated Score and Nomogram Combining Clinical and Immunohistochemistry Factors to Predict High ISUP Grade Clear Cell Renal Cell Carcinoma

**DOI:** 10.3389/fonc.2018.00634

**Published:** 2018-12-18

**Authors:** Junlong Wu, Wen-Hao Xu, Yu Wei, Yuan-Yuan Qu, Hai-Liang Zhang, Ding-Wei Ye

**Affiliations:** ^1^Department of Urology, Fudan University Shanghai Cancer Center, Shanghai, China; ^2^Department of Oncology, Shanghai Medical College, Fudan University, Shanghai, China

**Keywords:** ISUP grade, renal tumor biopsy, prediction model, immunohistochemistry, clear cell renal cell carcinoma

## Abstract

**Objective:** The International Society of Urological Pathology (ISUP) has proposed a grading system to classify renal cell carcinoma (RCC). However, classification using biopsy specimens remains problematic and, consequently, the accuracy of a biopsy-based diagnosis is relatively poor. This study aims to combine clinical and immunohistochemical (IHC) factors for the prediction of high ISUP grade clear cell RCC (ccRCC) in an attempt to complement and improve the accuracy of a biopsy-based diagnosis.

**Methods:** A total of 362 ccRCC patients were enrolled in this study and used for the training set. We performed IHC analysis of 18 protein markers on standard tissue sections using an automated stainer. Multivariate logistic regression models were developed to evaluate independent predictors for high ISUP grade. We evaluated different prediction models using receiver operating characteristic (ROC) curves and area under the ROC curve (AUC) analysis. A nomogram for the derivation of an integrated score for predicting high ISUP grade ccRCC and a calibration curve were also plotted. Finally, an internal validation cohort was examined to evaluate the performance of our integrated scoring system and nomogram.

**Results:** Multivariate logistic analyses revealed seven credible candidates for predicting high grade ISUP. These were age, tumor diameter, surgery, and CK7, Ki-67, PTEN, and MTOR protein expression. The ROC curves for the clinical, IHC and integrated models were compared in the training set, and the AUC for each was 0.731, 0.744, and 0.801, respectively. DeLong's test showed that the integrated model was significantly better at predicting high ISUP grade, when compared with the other models. Internal validation confirmed the good performance of the integrated score in predicting ISUP grade.

**Conclusion:** We have developed a nomogram integrating clinical and immunohistochemical parameters to predict high ISUP grade for M0 ccRCC patients. This nomogram may offer potentially useful information during preoperative individualized patient risk assessment, and consequently may help urologists when planning personalized management regimens.

## Introduction

Renal cell carcinoma (RCC) is one of the most common malignancies of the genitourinary system, and constitutes 3% of adult malignant tumors and accounts for 2% of all cancer mortality (1). Clear cell RCC (ccRCC) is the predominant histological subtype of RCC, accounting for approximately 80% of cases (2). Advanced ccRCCs are thought to be associated with a more aggressive clinical course and worse prognosis (3). The Fuhrman system (1982) was originally recommended as the histological grading system for RCC (4). Although widely used, it failed to take into account the latest histologic subtypes of RCC, and was prone to poor interpretability, and consequently poor inter-observer concordance regarding histopathological scoring (5). In 2012, the International Society of Urological Pathology (ISUP) Consensus Conference made recommendations regarding prognostic characteristics, staging, and immunohistochemical (IHC) evaluation and classification, in an attempt to improve the prognostic value of these factors in the assessment of the various histomorphological phenotypes of RCC (6, 7). By applying these recommendations, increased tumor grade was found to be significantly associated with poor patient outcomes (8). Therefore, in 2016 the International Agency for Research on Cancer replaced the Fuhrman system with a new grading standard based on the WHO/ISUP grading system, which is summarized in Supplementary Table S1 (9, 10).

The histologic subtype and ISUP grade of RCC are commonly based on the pathological diagnosis of surgical specimens (10). Alternatively, for patients with a small renal mass, percutaneous renal tumor biopsy (RTB) may also facilitate the pathological diagnosis and even the evaluation of nuclear grade. RTB also plays an important role in the screening of candidates for personalized therapy, and yet the sensitivity and accuracy of RTB analysis remains questionable (11), particularly for ccRCC (12–15). Insufficient biopsy tissue and tumor heterogeneity may both lead to inaccuracy in ISUP grading (16, 17). In 2016, a systematic review and meta-analysis by Marconi et al. suggested that the diagnostic accuracy and safety of RTB in RCC should be affirmed, although conclusive evidence or definitive guidelines for this approach remain lacking (18).

Accuracy of diagnosis from RTB remains inadequate; biopsy analysis is associated with a 14.1% non-diagnostic rate, and 90.4% of these are surgically confirmed malignancies (19). Using RTB to define histologic subtype is as precise as using surgical specimens, but inconsistencies exist in the classification of nuclear grade (consistency rate of only 82%) (20). Given the diagnostic inadequacies associated with preoperative biopsy in ccRCC, an increasing number of studies have attempted to incorporate more convenient clinical profiles and IHC or gene expression biomarkers to refine the ISUP grading system (21–23).

In this study, we investigated the value of integrating clinical profiles and IHC biomarkers in facilitating the diagnosis of high ISUP grade ccRCC. A total of 362 patients undergoing radical nephrectomy or nephron-sparing surgery (NSS) in our institute were recruited for the study. Our analysis was used to construct a nomogram for the prediction of ISUP grade risk in ccRCC and we propose that this nomogram has potential utility in guiding patient management decisions. In addition, variations in the expression of IHC markers observed in our analysis may inspire new research aimed at further refining the renal ISUP grading system.

## Materials and Methods

### Ethics Statement

Our study was approved by The Clinical Research Ethics Committee of the Fudan University Shanghai Cancer Center. Written informed consents were obtained from all subjects enrolled in this study. Study design and all testing procedures were performed according to the ethical standards of the Helsinki Declaration II.

### Study Population

Our training cohort consisted of patients with M0 ccRCC who underwent radical nephrectomy or NSS between Dec 2015 and Jun 2017. For the internal validation cohort, we included ccRCC patients who underwent surgery between Jul 2017 and Apr 2018 and who had full documentation for their pre-operative surgical plan. Each hematoxylin and eosin slide was reviewed independently by two experienced pathologists to determine the accuracy of the pathological diagnosis. Clinicopathological characteristics including age of onset, body mass index (BMI), gender, tumor location, ISUP grade, and TNM stage were obtained from our medical records or from pathology reports.

### IHC Staining and Evaluation

Immunohistochemistry was performed on standard tissue sections using an automated IHC stainer (Ventana, Tucson, AZ). In this study, we included 18 IHC markers. The primary antibodies used for the detection of these 18 proteins were as follows: anti-PAX-8 (Clone MRQ-50, Ventana), anti-P504S (Clone SP116, Ventana), anti-Ki-67 (Clone 30-9, Ventana), anti-CD10 (Clone SP67, Ventana), anti-HER2 (Clone 4B5, Ventana), anti-PTEN (Clone SP218, Ventana), anti-COX2 (Clone SP21, Ventana), anti-Vimentin (Clone Vim 3B4, Ventana), anti-TFE3 (Clone MRQ-37, Ventana), anti-CA9 (Cat No. 5649, Cell Signaling Technology), anti-CD117 (Cat No. 37805, Cell Signaling Technology), anti-mTOR (Cat No. 2983, Cell Signaling Technology), anti-CK7 (Cat No. ab181598, Abcam), anti-BAP1 (Cat No. ab199396, Abcam), anti-HGF (Cat No. ab83760, Abcam), anti-SETD2 (Cat No. PA5-43071, Invitrogen), anti-HIF-1α (Cat No. MA1-516, Invitrogen) and anti-PBRM1 (Cat No. HPA015629, Sigma-Aldrich). Criteria for the positive and negative scoring of IHC specimens for a given protein marker has been described previously (24), and all samples were evaluated by two independent experienced pathologists.

### Statistical Analysis

Patients were classified as symptomatic at the time of renal tumor diagnosis if they presented with hematuria, a palpable abdominal mass, or waist/back pain. Gender, symptoms, tumor location, surgery, pathological T (pT) stage, pathological N (pN) stage, hypertension, were treated as categorical variables and presented as proportions. Age at diagnosis, BMI and maximal tumor diameter were treated as continuous variables and reported as means with standard deviation (SD). TNM staging was in accordance with the 7th AJCC TNM 2010 system (25). ISUP grading followed the recommendations of the 2012 ISUP Consensus Conference for the Classification, Grading and Staging of Renal Neoplasia (5).

Normally distributed continuous data were compared using the Student's *t*-test. The Chi-square test was used to compare the distribution of categorical data between groups. Univariate and multivariate regression models were developed to find independent predictors, including clinical factors and IHC-related factors, for high ISUP grade ccRCC. All tests were two-tailed and *P*-values < 0.05 were considered statistically significant.

### Evaluation of Nomogram Performance

The receiver operating characteristic curve (ROC) was constructed by predicting the probability of a diagnosis being of low or high ISUP grade risk. ROC curves were plotted with confidence intervals and AUC analysis was performed to determine the best prediction model. DeLong's non-parametric test was used compare AUC data and evaluate model performance. A nomogram of integrated scores for predicting high ISUP grade ccRCC and a calibration curve were also plotted. All statistical analysis and graphical plotting were conducted using R software.

## Results

This study consisted of two stages. In the first stage, associations between specific clinical or IHC markers and ISUP grade were assessed; in the second stage, a prognostic model comprising combined profiles was constructed and verified to predict high grade risk using a nomogram.

### Clinical Characteristics of Patients Classified According to ISUP Grade

Data from 362 patients with ccRCC who met the inclusion criteria were included in the analyses. The medical records of patients were retrospectively reviewed. Each subgroup was classified according to ISUP grade, with 68.2% (*N* = 247) of cases assigned to the low ISUP grade (I–II) group and 31.8% (*N* = 115) of cases assigned to the high ISUP grade (III–IV) group. Clinical and pathological parameters included age, BMI, maximal tumor diameter, gender, symptoms, tumor location, surgical method, TNM stage, hypertension, diabetes, cardiovascular disease, and personal cancer history, and are summarized in Table 1.

**Table 1:** Localized clear cell renal cell carcinoma patients' characteristics in FUSCC.

**Characteristics**	**Entire cohort (*N* = 362)**	**Sub-groups classified by ISUP grade**		
		**ISUP grade I–II (*N* = 247)**	**ISUP grade III–IV (*N* = 115)**	***P*-value**
**MEAN (SD)**				
Age, years	55.78 (10.97)	54.50 (10.91)	58.54 (10.62)	**0.001**^a^
BMI, kg/m^2^	24.30 (3.32)	24.27 (3.48)	24.36 (2.97)	0.806^a^
Maximal tumor diameter, cm	3.80 (1.99)	3.36 (1.82)	4.73 (2.05)	**< 0.001**^a^
**N (%)**				
Gender				0.190^b^
Male	254 (70.2)	168 (68.0)	86 (74.8)	
Female	108 (29.8)	79 (32.0)	29 (25.2)	
Symptom				**0.009**^b^
No	271 (74.9)	195 (78.9)	76 (66.1)	
Yes	91 (25.1)	52 (21.1)	39 (33.9)	
Location				0.114^b^
Left kidney	170 (47.0)	109 (44.1)	61 (53.0)	
Right kidney	192 (53.0)	138 (55.9)	54 (47.0)	
Surgery				**<0.001**^b^
Radical nephrectomy	108 (29.8)	50 (20.2)	58 (50.4)	
Nephron-sparing	254 (70.2)	197 (79.8)	57 (49.6)	
pT stage				0.108^b^
T1 – T2	341 (94.2)	236 (95.5)	105 (91.3)	
T3 – T4	21 (5.8)	11 (4.5)	10 (8.7)	
pN stage				0.473^c^
N0	353 (97.5)	242 (98,0)	111 (96.5)	
N1	9 (2.5)	5 (2.0)	4 (3.5)	
Hypertension				0.825^b^
No	239 (66.0)	164 (66.4)	75 (65.2)	
Yes	123 (34.0)	83 (33.6)	40 (34.8)	
Diabetes				0.678^b^
No	314 (86.7)	213 (86.2)	101 (87.8)	
Yes	48 (13.3)	34 (13.8)	14 (12.2)	
Cardiovascular disease				0.979^b^
No	324 (89.5)	221 (89.5)	103 (89.6)	
Yes	38 (10.5)	26 (10.5)	12 (10.4)	
Personal cancer history				0.113^c^
No	350 (96.7)	236 (95.5)	114 (99.1)	
Yes	12 (3.3)	11 (4.5)	1 (0.9)	

a *Student's t-test*.

b *Chi-square test*.

c *Fisher exact test*.

*P-value < 0.05 is highlighted using bold font*.

Patients with a high ISUP grade were more likely to be older (*P* = 0.001), have a larger maximal tumor diameter (*P* < 0.001), be symptomatic (*P* = 0.009), and to have undergone radical nephrectomy (*P* < 0.001).

### Performance of Clinical, IHC and Combined Markers in Outcome Prediction

In multivariate logistic regression models, maximal tumor diameter was associated with high ISUP grade in patients with ccRCC, with an odds ratio [OR; 95% confidence interval (CI)] of 1.264 (1.083–1.476, *p* = 0.003). Additionally, age and surgical method (ref. NSS) were clear predictors of high ISUP grade subgroups risk with ORs of 1.026 (1.003–1.050, *P* = 0.026) and 2.103 (1.155–3.829, *P* = 0.015), respectively (Table 2).

**Table 2:** Univariate and multivariate logistic regression analysis of clinical factors in predicting high ISUP grade.

**Clinical factors**	**Univariate analysis**			**Multivariate analysis**		
	**OR**	**95% CI**	***P*-value**	**OR**	**95% CI**	***P*-value**
Age, years	1.036	1.014–1.058	**0.001**	1.026	1.003–1.050	**0.026**
BMI, kg/m^2^	1.008	0.944–1.078	0.806			
Maximal tumor diameter, cm	1.441	1.264–1.643	**<0.001**	1.264	1.083–1.476	**0.003**
Gender (ref. Male)	0.717	0.436–1.181	0.191			
Symptom (ref. None)	1.924	1.176–3.149	**0.009**	1.439	0.838–2.471	0.188
Location (ref. Left)	0.699	0.448–1.090	0.114			
Surgery (ref. NSS)	4.016	2.481–6.494	**<0.001**	2.103	1.155–3.829	**0.015**
pT stage (ref. T1-2)	2.043	0.842–4.959	0.114			
pN stage (ref. N0)	1.744	0.460–6.620	0.414			
Hypertension (ref. None)	1.054	0.661–1.679	0.825			
Diabetes (ref. None)	0.868	0.446–1.690	0.678			
Cardiovascular disease (ref. None)	0.990	0.481–2.040	0.979			
Personal cancer history (ref. None)	0.188	0.024–1.476	0.112			

*P-value < 0.05 is highlighted using bold font*.

**Figure 1:**
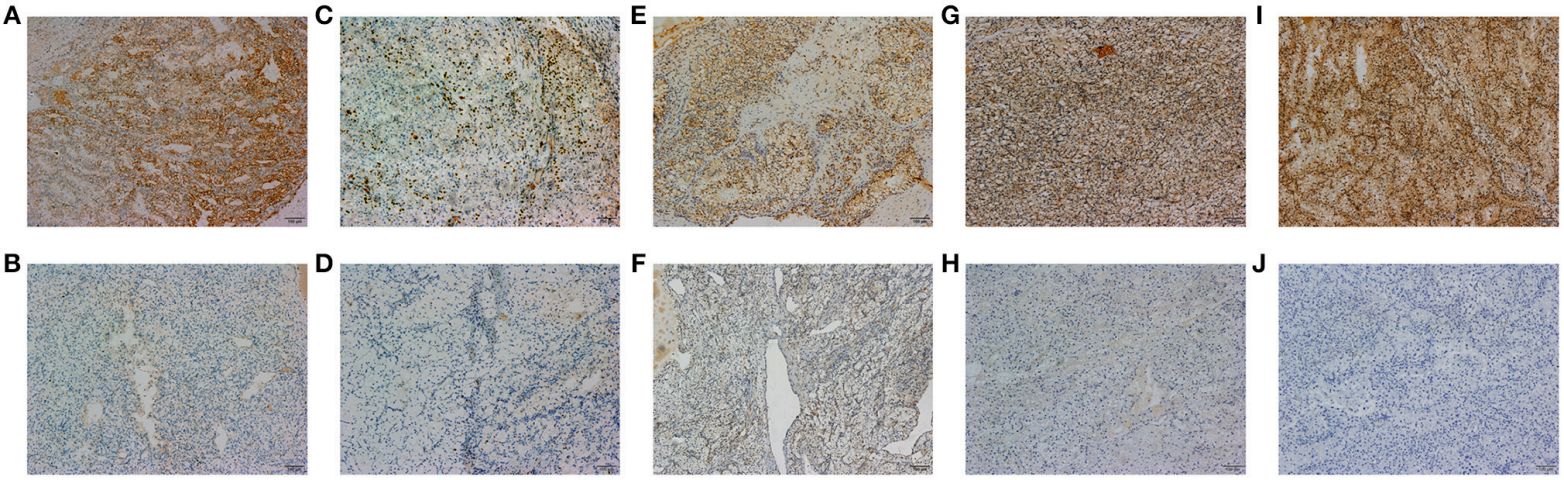
The representative images of CK7 **(A,B)**, Ki-67 **(C,D)**, PTEN **(E,F)**, MTOR **(G,H)**, and HIF-1α **(I,J)** positive and negative IHC staining of tumor tissues are shown in 100x standard microscopic enlargement.

A total of 18 IHC markers were assessed in the 362 patient cohort. As shown in Table 3, for localized RCC patients, multivariate regression analysis suggested that five of the 18 protein markers were independent predictors of high ISUP grade. These were CK7 [OR, 95%CI (0.493, 0.250–0.975)], Ki-67 (2.806, 1.682–4.681), PTEN (2.702, 1.241–3.459), mTOR (0.452, 0.251–0.816), and HIF-1α (0.503, 0.276–0.917). Representative images of positive and negative IHC standing for these five predictive protein markers are shown in Figure 1.

**Table 3:** Univariate and multivariate logistic regression analysis of IHC markers in predicting high ISUP grade.

**IHC markers (ref. Low expression)**	**Univariate analysis**			**Multivariate analysis**		
	**OR**	**95% CI**	***P*-value**	**OR**	**95% CI**	***P*-value**
CA9	0.933	0.275–3.163	0.911			
CK7	0.325	0.180–0.587	**<0.001**	0.493	0.250–0.975	**0.042**
PAX-8	0.562	0.334–0.948	**0.031**	0.772	0.434–1.374	0.380
P504S	1.795	0.676–4.769	0.241			
Ki-67^a^	3.628	2.282–5.769	**<0.001**	2.806	1.682–4.681	**<0.001**
CD10	–	–	0.999			
HER2	–	–	0.999			
PTEN	1.899	1.201–3.004	**0.006**	2.072	1.241–3.459	**0.005**
COX-2	0.650	0.170–2.490	0.530			
Vimentin	0.609	0.231–1.605	0.316			
BAP1	0.841	0.464–1.526	0.569			
CD117	1.740	0.392–7.723	0.466			
HGF	0.912	0.473–1.759	0.784			
SETD2	0.827	0.507–1.351	0.448			
TFE3	1.168	0.457–2.982	0.745			
HIF-1α	0.394	0.235–0.661	**<0.001**	0.503	0.276–0.917	**0.025**
MTOR	0.529	0.316–0.886	**0.016**	0.452	0.251–0.816	**0.008**
PBRM1	1.158	0.576–2.329	0.681			

a *Positive rate ≥10% was defined as high expression*.

*P-value < 0.05 is highlighted using bold font*.

After reintegrating all of the significant clinical and IHC indicators in the logistic regression model, multivariate analysis suggested that HIF-1α was of no statistically significant value in predicting grade (0.567, 0.296–1.086) and was therefore excluded from the final panel. The remaining seven indicators identified as credible candidates included age (0.026, 1.003–1.054), tumor diameter (1.281, 1.091–1.504), surgical method (2.088, 1.098 to −3.968), and CK7 (0.356, 0.167–0.760), Ki-67 (2.672, 1.535–4.650), PTEN (1.960, 1.130–3.400), and mTOR (0.483, 0.256–0.909) expression (Table 4).

**Table 4:** Multivariate logistic regression analysis of combined markers in predicting high ISUP grade.

	**Multivariate analysis**		
	**OR**	**95% CI**	***P*-value**
**CLINICAL MARKERS**			
Age, years	1.028	1.003–1.054	**0.026**
Maximal tumor diameter, cm	1.281	1.091–1.504	**0.003**
Surgery (ref. NSS)	2.088	1.098–3.968	**0.025**
**IHC MARKERS (REF. LOW EXPRESSION)**			
CK7	0.356	0.167–0.760	**0.008**
Ki-67^a^	2.672	1.535–4.650	**0.001**
PTEN	1.960	1.130–3.400	**0.017**
HIF-1α	0.567	0.296–1.086	0.087
MTOR	0.483	0.256–0.909	**0.024**

a *Positive rate ≥10% was defined as high expression*.

*P-value < 0.05 is highlighted using bold font*.

### Construction of a Nomogram Integrating Both Clinical and IHC Biomarkers

**Figure 2:**
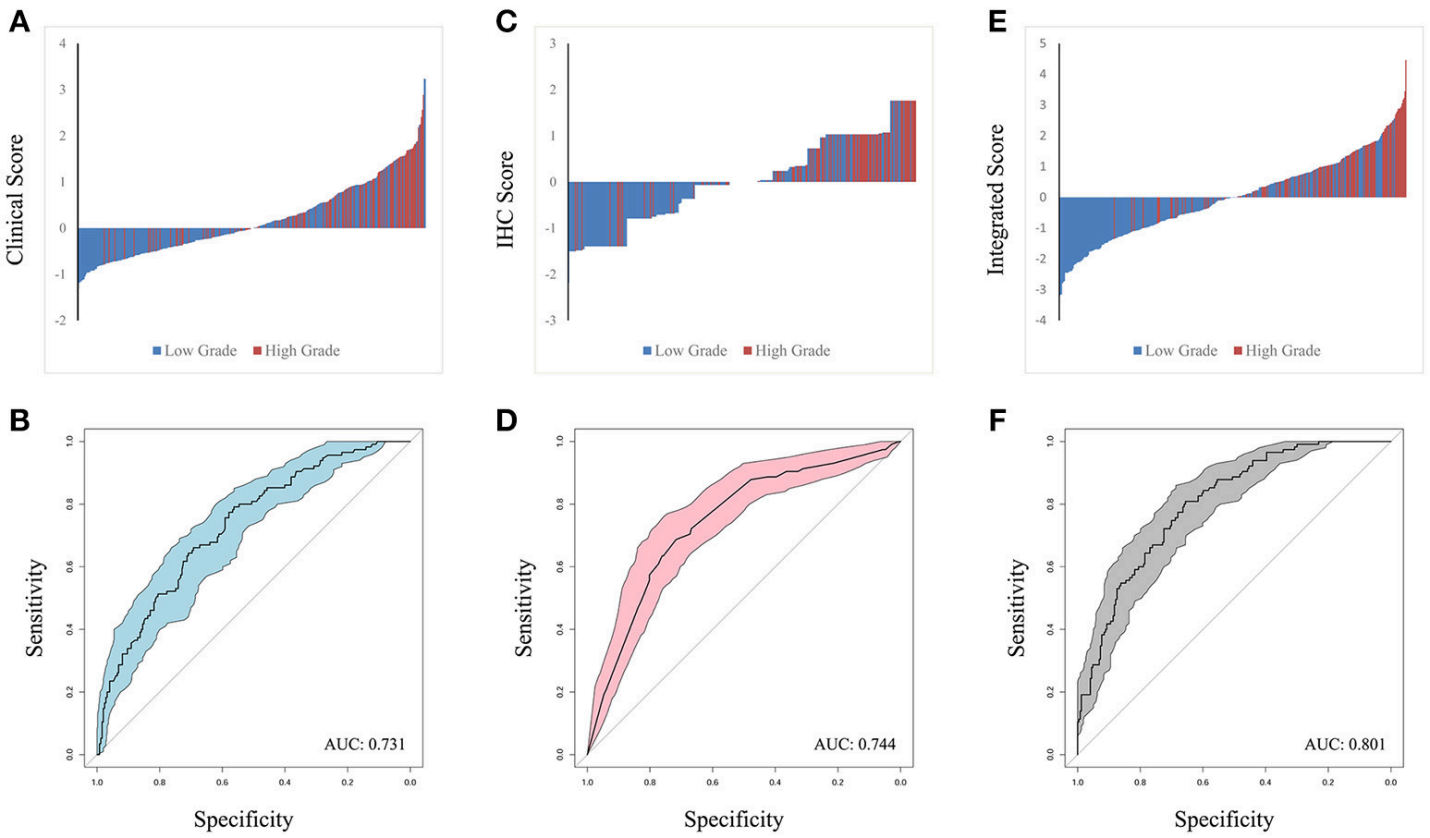
Waterfall plot of different models was contrasted in clinical factors **(A)**, IHC markers **(C)** and integrated penal **(E)**, with horizontal axis representing the patients and vertical axis the score. ROC curve were performed to validate low or high ISUP classification from based on the three logit models. The shadow part represent confidential interval and AUC index in clinical, IHC and integrated indicators was 0.731, 0.744, and 0.801 in **(B,D,F)**, respectively.

The predictors identified in the independent and integrated multivariate logistic regression analysis were used to construct three prediction models based on clinical, IHC or integrated clinical/IHC data and the predictive value of each model was subsequently evaluated. Waterfall plots for each of the three models are shown in Figures 2A,C,E. In addition, ROC curves were generated to validate the ability of each logistic model to predict low or high ISUP grade (Figures 2B,D,F). The shaded region of each curve represents the confidence interval. The AUC indices for the clinical, IHC and integrated indicators were 0.731, 0.744, and 0.801, respectively. To facilitate comparisons and to demonstrate visually the advantages of the integrated model, ROC curves for all three models are plotted together in Supplementary Figure S1. DeLong's test was used to assess concordance among the different prediction models, and showed that the integrated model performed better than both the clinical (*P* = 0.001) and IHC (*P* = 0.008) models in terms of predictive power. However, a statistically significant difference was not observed between the AUCs of the two separate-domain models (*P* = 0.744).

**Figure 3:**
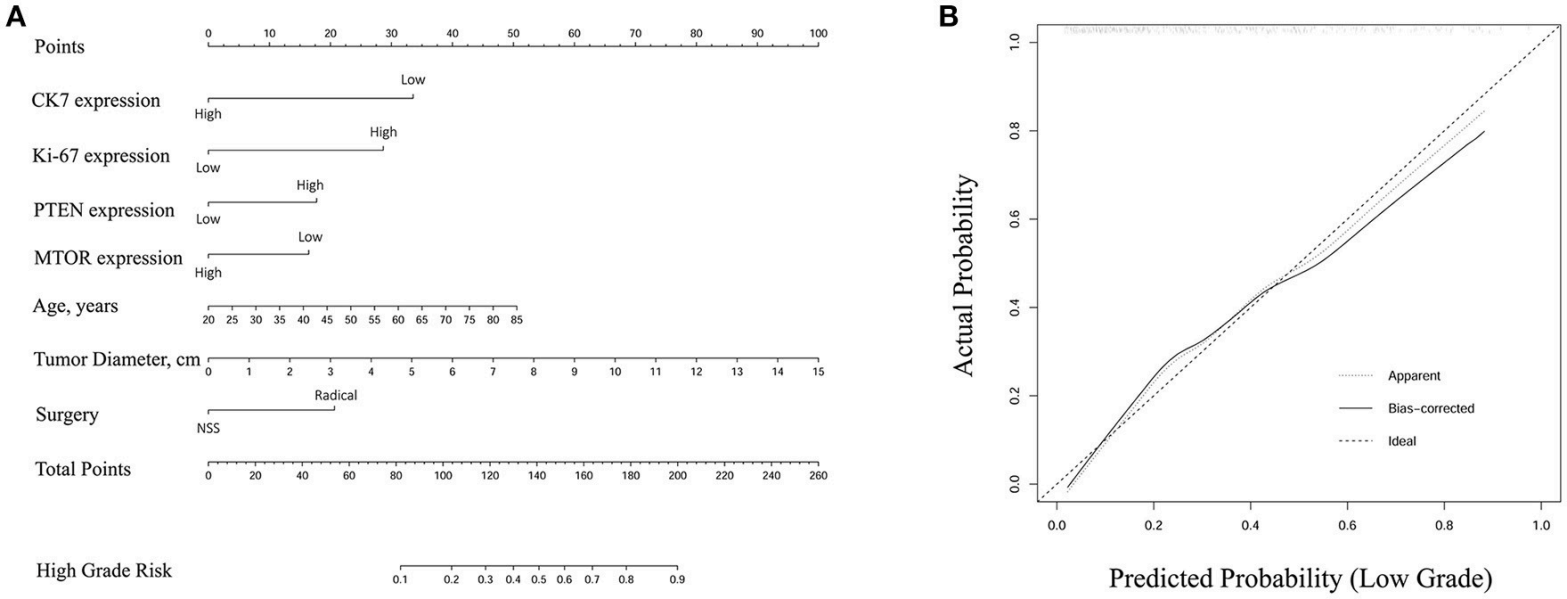
**(A)** Nomogram of integrated score for predicting high ISUP grade. The total points were conducted by summarizing the points for each variable. High grade risk was determined by specific total points at the bottom of plotting scale. **(B)** The calibration curve was closely consistent with ideal diagonal curve (*P* < 0.05), indicating that this nomogram was in high precision.

A nomogram of integrated scores for predicting high ISUP grade and a calibration curve are plotted in Figure 3. The nomogram is used as follows: for each variable, a vertical line is drawn from the relevant point on the given axis up to the “points” axis, and the score at the point of intersection is then recorded. This procedure is repeated for the six other variables and all scores are then summed to provide the total score. The total score is located on the total points axis and a vertical line then drawn down to the High Grade Risk axis to obtain the high ISUP grade risk. The calibration plot closely resembled the ideal diagonal curve (*P* < 0.05), indicating that the nomogram was of high precision.

### Internal Validation of the Integrated Nomogram for Predicting High ISUP Grade

**Figure 4:**
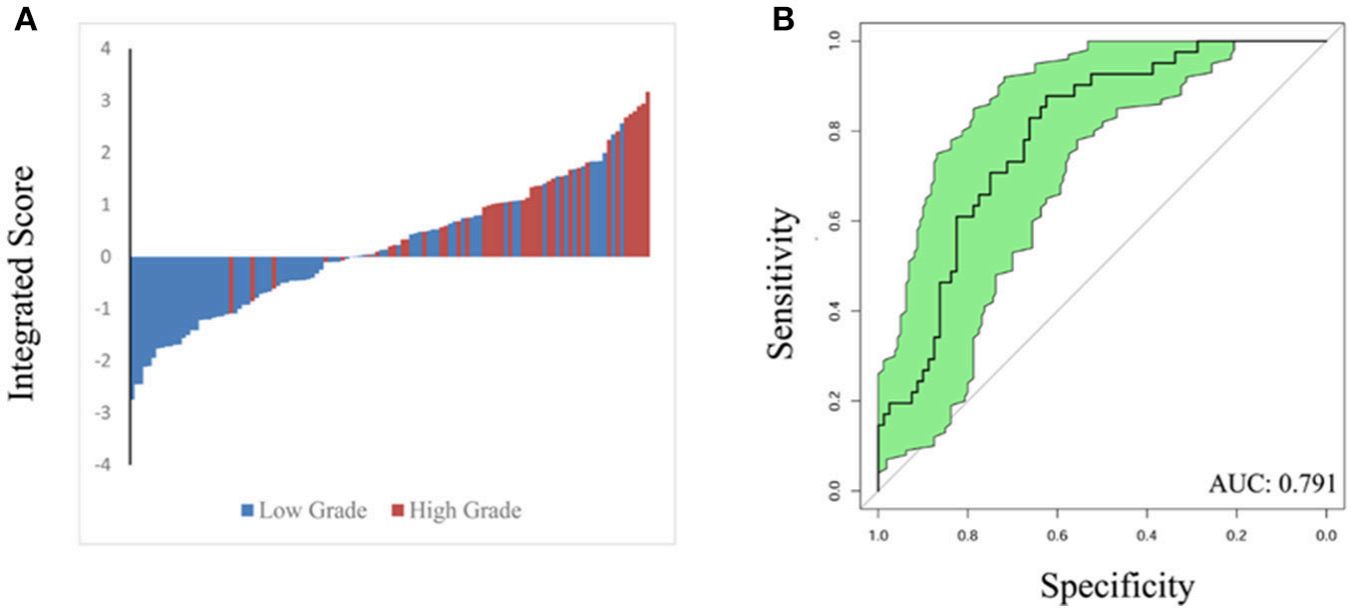
Waterfall plot **(A)** and ROC curve **(B)** were constructed to validate predicting performance based on integrated score and nomogram in internal validation set (121 patients). AUC index is 0.791.

To validate the performance of our integrated score and nomogram in predicting high ISUP grade, we recruited 121 ccRCC patients with fully recorded pre-operative surgical plans. For the internal validation set, we used “pre-operation surgical plan” as the “surgery” parameter in the integrated nomogram to test the predictive value of this integrated score in the clinical setting. A waterfall plot and ROC curve for the internal validation set are shown in Figure 4. The AUC index was 0.791, which was similar to that that of the training set. This analysis confirmed that the integrated score and nomogram demonstrate good performance in predicting ISUP grade based on pre-operative surgical plans and other parameters. Our system may therefore have potential clinical utility as a means of predicting high ISUP grade in ccRCC patients and informing subsequent patient management decisions.

## Discussion

In addition to the specific characteristics of a patient and the expertise of the urologist, individual cancer therapy is primarily based on an assessment of the biological characteristics of the tumor. Although appropriate radiological examinations are widely used to assess TNM stage, classification of tumor grade provides an additional useful tool in guiding clinicians toward appropriate treatment regimens. The identification of prognostic factors such as ISUP grade may in turn help clinicians identify malignancies that are suitable for specific treatments. However, accurate ISUP grading from biopsy samples is frequently hindered by tumor heterogeneity and insufficiencies in the amount of the harvested material. In the current study, a practical method was adopted to identify potential factors that serve as predictors of high ISUP grade risk. A total of seven factors were identified, including age, tumor diameter, surgery, and CK7, Ki-67, PTEN, and mTOR expression, and these factors were integrated into a model for the prediction of high grade risk. The accuracy of this integrated model was significantly higher than that of models based on clinical or IHC factors alone. It is noteworthy that all of the predictive variables included in the model are easily obtainable in a single clinical center, and consequently our nomogram may serve as a supplement to enhance the accuracy of a conventional RTB-based pathologic diagnosis. As far as we know, this is the first integrated predictive model for ISUP grade prediction in ccRCC. This nomogram potentially provides a practical tool for clinical risk assessment and could therefore form the basis of personalized risk-adaptive therapy.

For patients with a small renal mass, RTB is commonly performed to identify nuclear morphology before treatment by cryotherapy or ablation. In 2012, the diagnostic accuracy rate of RTB as a histologic procedure for differentiating between malignant and benign tumors was reported to be approximately 60% (26). Likewise, one biopsy strategy for Chinese patients has indicated that using RTB to define histologic subtype is as accurate as using surgical specimens with a consistency rate as high as 82% (although nuclear grade classification is less consistent) (20). Recently, the European Association of Urology (EAU) recommended that all patients under active surveillance undergo biopsy, while the American Urological Association (AUA) has suggested RTB as an alternative option for such patients. Furthermore, the EAU also considers RTB to be an accurate and safe diagnostic method for evaluating malignancy and histological subtype. However, the systematic review was not of a high quality. In our study, the integrated panel, which is composed of both clinical and IHC variables, significantly increased the accuracy of nuclear grading (AUC: 0.801). Indeed, our integrated model has better predictive ability than subjective assessment or reliance on independent indicators such as tumor size, AJCC stage and necrosis (27). Therefore, the nomogram may help to increase the sensitivity of a histology-based diagnosis from RTB samples and, consequently, may improve the accuracy of ISUP grading before definitive treatment decisions are made.

Along with the development of new treatment options and targeted therapy, cancer subtype classification has become increasingly important in guiding personalized treatment strategies. In recent years, a variety of biomarkers have been identified that are associated with the different molecular subtypes of various carcinomas (28–30). This approach enables us to gain a more thorough understanding of ccRCC at the histological level. TNM stage and ISUP grade form the basis of current predictive or prognostic models. Hence, to avoid the over-diagnosis and excessive treatment of ccRCC patients caused by an analysis of poor-quality RTB samples, an improved approach for predicting ISUP grade that can better inform treatment decisions is required.

Clinical characteristics incorporated into our predictive nomogram included age and tumor size, which have already been recognized as prognostic factors for ccRCC (31). Interestingly, we also included surgery in our integrated nomogram constructed for the training set. Clearly, if patients have already had surgery, there is no need to have a tool for predicting grade because a pathologic specimen has already been obtained. Consequently, to improve the clinical application of the model, we used “pre-operative surgical plan” as the “surgery” parameter in our nomogram constructed for the internal validation set. With this substitution, the model demonstrate good performance in predicting high ISUP grade, indicating that a pre-operative surgical plan represents both a valid and useful variable. Importantly, to provide a measure of consistency, we also determined the likelihood that an established pre-operative surgical plan resulted in actual surgery in a cohort of 324 patients, and found that surgery consistency presented in 96.9% of cases (Supplementary Table S2).

IHC-based biomarkers are widely used to evaluate both tumor aggressiveness and patient prognosis in ccRCC (29). In this study, the IHC score provides some insight into the possible underlying reason for the predictive value of the ISUP grading system (AUC: 0.744). CK7, encoded by the KRT7 gene, is specifically expressed in the simple epithelia that lines the cavities of the internal organs, the gland ducts and the blood vessels (32, 33). CK7 expression was observed in both healthy and neoplastic cells and CK7 staining has prognostic value in an IHC-based analysis of renal cell neoplasms (34, 35). In addition, both chromophobe RCC and clear cell papillary RCC are typically positive for CK7, while conventional ccRCC is usually not, which grants it pivotal importance in the differential diagnosis among clear cell renal neoplasms (36). In 2018, Gonzalez et al. suggested that CK7 immunoreactivity in ccRCC is variable and the extent of staining depends on the clinical-histopathological parameters including grade and architectural growth patterns of the tumors (37). Ki-67 is a marker of cellular proliferation and is also involved in ribosomal RNA transcription (38). Its inactivation results in the inhibition of ribosomal RNA synthesis (39). In this study, Ki-67 status was considered as positive when ≥10% of cells demonstrated Ki-67 staining (40). The *PTEN* gene acts as a common tumor suppressor through the actions of its phosphatase protein product (41). This phosphatase is implicated in the regulation of the cell cycle and, consequently, cell proliferation (42). mTOR is a master regulator of cell growth and proliferation, and is also implicated in the control of cell motility, cell survival, protein synthesis, autophagy, and transcription (43–45). Activation of the mTOR signaling pathways results in deregulated protein synthesis and energy metabolism in many cancers (46). While the expression of each of the above protein markers has been thoroughly documented in a number of kidney cancer studies, the application of their combined expression profile in the prediction of high-grade ccRCC remains underexplored (23, 29).

Our study represents the first attempt to construct an integrated model from combined clinical and IHC factors to predict high ISUP grade risk and one of its main strengths lies in the use of specimens routinely obtained during the assessment of ccRCC patients. An additional strength lies in the fact that the seven parameters used to construct our model are relatively easy to obtain in the clinical setting, when compared with the parameters required for more traditional multi-gene prediction models (47).

However, there are also some limitations associated with our study. These include the use of a retrospective data set, the relatively small sample size, poor variation in the sample population, and possible selection bias associated with a single cancer center. Also, our integrated model requires future external validation in preoperative biopsy patient cohorts to confirm its value in improving the diagnostic accuracy of RTBs. Last, this study only included the 18 IHC markers that are routinely evaluated in our own cancer center, and this may therefore result in selection bias that decreases the predictive value of the model.

## Conclusion

We have developed an integrated nomogram comprising clinical and immunohistochemical indicators to predict high ISUP grade for M0 ccRCC patients. This nomogram offers potentially useful information during preoperative risk assessment, and may help urologists when evaluating personalized management regimens.

## Availability of Data and Material

The datasets during and/or analyzed during the current study available from the corresponding author on reasonable request.

## Author Contributions

The work presented here was carried out in collaboration among all authors. YW and H-LZ defined the research theme, discussed analyses, interpretation, and presentation. W-HX and JW drafted the manuscript, analyzed the data, developed the algorithm and interpreted the results. Y-YQ participated in IHC staining, co-worked on associated data collection and helped to draft the manuscript. D-WY helped to evaluated IHC profiles and performed the statistical analysis. All authors read and approved the final manuscript.

### Conflict of Interest Statement

The authors declare that the research was conducted in the absence of any commercial or financial relationships that could be construed as a potential conflict of interest.

## Supplementary Material

Supplementary Figure S1 ROC curves of three models were synchronously plotted to predict diagnosis probability. Blue line represents clinical score with AUC of 0.731. Red line represents IHC score with AUC of 0.744. Black line represents integrated score with AUC of 0.801.

Supplementary Table S1 Fuhrman grading system and updated World Health Organization/International Society of Urological Pathology (WHO/ISUP) grading system for clear cell renal cell carcinoma and papillary renal cell carcinoma

Supplementary Table S2 Consistency evaluation of pre-operation surgical plan and actual surgery.

## Abbreviations

RCC: renal cell carcinoma
ccRCC: clear cell renal cell carcinoma
ISUP: International Society of Urological Patheology
RTB: renal tumor biopsy
IHC: immunohistochemical
NSS: nephron-sparing surgery
ROC: receiver operating characteristic
AUC: area under the ROC curve
FUSCC: Fudan University Shanghai Cancer Center.
