# Supplementary figures and images for: An Integrated Score and Nomogram Combining Clinical and Immunohistochemistry Factors to Predict High ISUP Grade Clear Cell Renal Cell Carcinoma
Source: Front Oncol. 2018 Dec 18;8:634. doi: 10.3389/fonc.2018.00634 (PMC6305456; doi:10.3389/fonc.2018.00634)

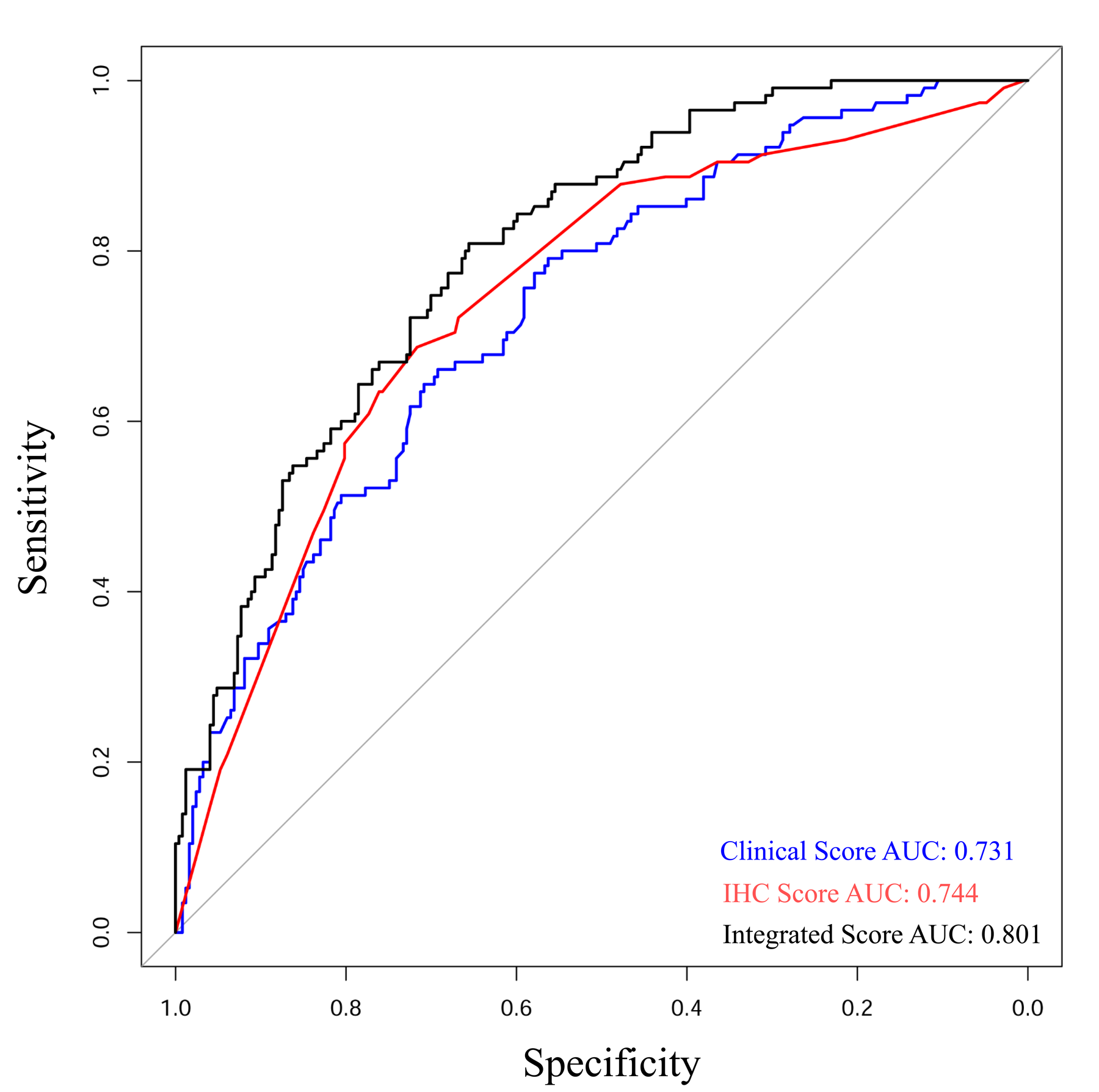

Supplement: Supplementary Figure S1 — ROC curves of three models were synchronously plotted to predict diagnosis probability. Blue line represents clinical score with AUC of 0.731. Red line represents IHC score with AUC of 0.744. Black line represents integrated score with AUC of 0.801. [file Image_1.TIF]
