# Supplementary material for: An Integrated Score and Nomogram Combining Clinical and Immunohistochemistry Factors to Predict High ISUP Grade Clear Cell Renal Cell Carcinoma
Source: Front Oncol. 2018 Dec 18;8:634. doi: 10.3389/fonc.2018.00634 (PMC6305456; doi:10.3389/fonc.2018.00634)
Supplement: Supplementary Table S1 — Fuhrman grading system and updated World Health Organization/International Society of Urological Pathology (WHO/ISUP) grading system for clear cell renal cell carcinoma and papillary renal cell carcinoma [file Table_1.DOCX]

**Supplementary Table 1. Fuhrman grading system and updated World Health Organization/International Society of Urological Pathology (WHO/ISUP) grading system for clear cell renal cell carcinoma and papillary renal cell carcinoma**

| **Grade Level** | **Nuclear Characteristics** | |
| --- | --- | --- |
|  | **Fuhrman grading system** | **WHO/ISUP grading system** |
| **G1** | **Nuclei appear round and uniform, 10 μm; nucleoli are inconspicuous or absent** | **Nucleoli are absent or inconspicuous and basophilic at x400 magnification** |
| **G2** | **Nuclei have an irregular appearance with signs of lobe formation, 15 μm; nucleoli are evident** | **Nucleoli are conspicuous and eosinophlic at x400 magnification and visible but not prominent at x100 magnification** |
| **G3** | **Nuclei appear very irregular, 20 μm; nucleoli are large and prominent** | **Nucleoli are conspicuous and eosinophlic at x100 magnification** |
| **G4** | **Nuclei appear bizarre and multilobated, 20 μm or more; nucleoli are prominent** | **There is pronounced nuclear pleomorphism, multinucleate giant cells, and/or rhabdoid and/or sarcomatoid differentiation** |
