# Supplementary material for: An Integrated Score and Nomogram Combining Clinical and Immunohistochemistry Factors to Predict High ISUP Grade Clear Cell Renal Cell Carcinoma
Source: Front Oncol. 2018 Dec 18;8:634. doi: 10.3389/fonc.2018.00634 (PMC6305456; doi:10.3389/fonc.2018.00634)
Supplement: Supplementary Table S2 — Consistency evaluation of pre-operation surgical plan and actual surgery. [file Table_2.DOCX]

**Supplementary Table 2.** **Consistency evaluation of pre-operation surgical plan and actual surgery.**

| **Entire cohort (N=324)** |  | **Actual surgery (N)** | | **Total** | **Consistency (%)** |
| --- | --- | --- | --- | --- | --- |
|  |  | **NSS** | **Radical nephrectomy** |  |  |
| **Pre-operation surgical plan (N, %)** |  |  |  |  |  |
| **NSS** |  | **205** | **9** | **214** | **95.8%** |
| **Radical nephrectomy** |  | **1** | **109** | **110** | **99.1%** |
| **Total** |  | **206** | **118** | **324** | **96.9%** |
